# Predatory journals: What can we do to protect their prey?

**DOI:** 10.1371/journal.pmed.1004542

**Published:** 2025-04-02

**Authors:** Christine Laine, Dianne Babski, Vivienne C. Bachelet, Till W. Bärnighausen, Christopher Baethge, Kirsten Bibbins-Domingo, Frank Frizelle, Laragh Gollogly, Sabine Kleinert, Elizabeth Loder, João Monteiro, Eric J. Rubin, Peush Sahni, Christina C. Wee, Jin-Hong Yoo, Lilia Zakhama

**Affiliations:** 1 Annals of Internal Medicine, Philadelphia, Pennsylvania, United States of America; 2 User Services and Collection Division, National Library of Medicine, Bethesda, Maryland, United States of America; 3 Medwave, Santiago, Chile; 4 PLOS Medicine, Public Library of Science, San Francisco, California, United States of America; 5 Deutsches Ärzteblatt (German Medical Journal) and Deutsches Ärzteblatt International, Cologne, Germany; 6 JAMA and the JAMA Network, Chicago, Illinois, United States of America; 7 New Zealand Medical Journal, Auckland, New Zealand; 8 Bulletin of the World Health Organization, Geneva, Switzerland; 9 The Lancet, London, United Kingdom; 10 The BMJ, London, United Kingdom; 11 Nature Medicine, New York, New York, United States of America; 12 New England Journal of Medicine, Boston, Massachusetts, United States of America; 13 The National Medical Journal of India, New Delhi, India; 14 Journal of Korean Medical Science, Seoul, Korea; 15 La Tunisie Médicale, Tunis, Tunisia

## Abstract

In this Editorial, representatives of the International Committee of Medical Editors discuss the need for multi-stakeholder involvement to recognize and counter the actions of predatory journals.

A growing number of entities misrepresent themselves as scholarly journals for financial gain despite not meeting scholarly publishing standards [1,2]. As editors and members of the International Committee of Medical Journal Editors (ICMJE), we receive queries about these “predatory” or “pseudo” entities and are subject to their deception when they target our authors and reviewers. The number of predatory journals is difficult to accurately determine but was estimated at more than 15,000 in 2021 [3]. While the ICMJE Recommendations include warnings about predatory publishing [4], the Committee believes that the large number of increasingly bold predatory entities warrants shining a bright light on them and considering actions stakeholders can take to counter their deceptive efforts.

The practices that these entities employ include aggressive solicitation of manuscript submissions; the promise of extremely rapid turnaround times; and a lack of transparency about article submission, processing, and even withdrawal charges. Predatory journals may claim that they follow legitimate editorial and publishing practices but do not actually conduct peer review or such functions as archiving journal content, managing potential conflicts of interest, enabling corrections, and responding to author queries in a timely manner. In egregious cases, the “published” articles never appear despite authors having paid the requested fees.

Predatory journals often use journal names and branding features that mimic well-established journals. They may falsely state that they are members of or follow the recommendations of respected organizations such as the Committee on Publication Ethics, the Council of Science Editors, ICMJE, and others. Predatory journals may fabricate indexing and citation metrics or may even have fallen through the cracks in the vetting process and be indexed [5]. To lend a veneer of credibility, these entities solicit individuals to serve on their editorial boards or as guest editors, sometimes listing persons in these roles without their consent. Predatory entities engage in these practices to purposefully deceive authors into submitting their work and paying associated fees [6]. Profits rise with the number of authors whom the predatory journal successfully captures.

These deceptive practices endanger authors, academic institutions, legitimate journals, legitimate publishers, the scholarly publishing process, science, and the public [6]. Particularly vulnerable authors are those who are early in their careers, lack experience and adequate mentorship, and face pressure to publish. Publication in a predatory journal may result in financial and professional consequences that interfere with the ability to publish work in legitimate journals. It is damaging to institutions’ credibility when their faculty and grantees fall prey to these entities. Legitimate journals and publishers whom predatory entities mimic may receive unfounded accusations of improper behavior. The existence of cunning predatory journals makes some academics and their institutions wary of legitimate open access, author-pays journals. Importantly, predatory journals can facilitate the dissemination of unvetted, weak, or even fraudulent health information [7].

## What can authors do?

Authors must be aware that predatory journals exist and avoid submitting their work to them by evaluating the integrity of the journals they seek to publish in. Seeking the assistance of experienced mentors, colleagues, and librarians may be helpful. Unfortunately, no current, comprehensive, and accurate list of predatory journals is available. Creation of such a list is infeasible as new entities continuously appear and disappear. However, guidance from various organizations is available to help identify the characteristics of reputable peer-reviewed journals.

The World Association of Medical Editors offers practical recommendations that include a set of questions authors should ask when choosing a venue for publication [2]. The ThinkCheckSubmit.org site provides a checklist of features that can help authors identify trusted journals and publishers [8]. The site also includes a brief video about predatory publishing. In 2017, the U.S. National Institutes of Health issued guidance to help their funded researchers distinguish reputable journals from predatory journals [9]. Authors should become familiar with these resources. When they have concerns about a particular journal’s legitimacy, they should share those concerns with colleagues and their institutions.

Because predatory journals mimic legitimate entities, authors need to be vigilant when they receive a solicitation from a journal or publisher to submit their work or serve in an editorial role. They should carefully check the e-mail address and URLs included in the communication to see if they match those of the legitimate entity. They may also contact the legitimate journal, forwarding the solicitation to inquire whether it actually came from that journal. Doing so not only protects the author from engaging with a predatory journal but also alerts the legitimate journal that it is being imitated.

## What can institutions and funders do?

Academic institutions and funders should be invested in helping their constituents avoid predatory journals. They can achieve this by making the resources mentioned herein available via institutional channels such as training materials, especially to those early in their careers, and routinely reviewing where faculty and grantees publish. Institutional librarians are familiar with the journals that people at their institution read and seek to publish in and can play an important role in helping guide authors to legitimate journals. Like authors, librarians who become aware of concerns about a journal’s legitimacy should share that information with their constituents as well as with librarians at other institutions. When librarians see a predatory journal that appears to be imitating a legitimate journal or publisher, they should alert their institutions and the mimicked journal.

In some situations, authors under pressure to publish may knowingly choose to publish in suspect journals to build a long list of publications to support academic promotion. This strategy would not be as effective if academic promotion committees weigh not only the quantity but also the quality of publications and the journals in which they appear.

## What can journal editors and publishers do?

Journals should alert authors to the existence of predatory journals and the resources mentioned herein in their information for authors and in any “how to get published” programs they offer. If editors and publishers become aware of a predatory entity that is imitating them, they should consider alerting their author community by posting a message on their website or sending an e-mail communication to their authors, reviewers, and editorial board members. Editors should recognize that authors may cite articles in predatory journals and should alert authors when they have concerns about the legitimacy of a citation.

Legal action against the predators is challenging because the predatory publishers are often ghost entities, contact persons can be difficult to identify, and unresponsiveness to communication is common. However, publishers should still issue cease and desist letters because these actions can deter continued predatory behaviors even if no response is received.

Predatory journals have developed strategies to profit by taking advantage of a climate that nurtures the growth of open access, author-pays publication models. It is worrisome that despite the awareness of these entities for many years, academicians still fall prey to them. Protecting the scientific community and the public from predatory journals requires action by all stakeholders.

**Note:** This article is being simultaneously posted on ICMJE.org and published in Annals of Internal Medicine, The BMJ, Bulletin of the World Health Organization, Deutsches Ärzteblatt (German Medical Journal), JAMA, Journal of Korean Medical Science, The Lancet, La Tunisie Médicale, The National Medical Journal of India, Medwave, Nature Medicine, New England Journal of Medicine, New Zealand Medical Journal, and PLOS Medicine.

CL reports employment by American College of Physicians and that her spouse has stock options in Targeted Diagnostics and Therapeutics. TB reports grants from the National Institutes of Health (NIH), Alexander von Humboldt Foundation, German National Research Foundation, European Union, Germany Ministry of Education and Research, German Ministry of the Environment, Wellcome, and KfW; serving on scientific advisory boards for NIH-funded research projects in Africa on climate change and health; and small stock ownership (EUR 5000) in CHEERS, an SME focused on approaches to measure climate change and health in population cohorts. KB reports employment by the American Medical Association and relationships with Resolve to Save Lives and with University of California, San Francisco. EL reports employment by the British Medical Association. JM reports employment by Springer Nature. CW reports her role as Senior Deputy Editor of Annals Internal Medicine, employment by American College of Physicians, and teaching activities at Beth Israel Deaconess Medical Center, Harvard University, and Boston Medical Center for which she receives honoraria.
